# Identifying underlying medical causes of pediatric obesity: Results of a systematic diagnostic approach in a pediatric obesity center

**DOI:** 10.1371/journal.pone.0244508

**Published:** 2020-12-17

**Authors:** Lotte Kleinendorst, Ozair Abawi, Bibian van der Voorn, Mieke H. T. M. Jongejan, Annelies E. Brandsma, Jenny A. Visser, Elisabeth F. C. van Rossum, Bert van der Zwaag, Mariëlle Alders, Elles M. J. Boon, Mieke M. van Haelst, Erica L. T. van den Akker

The affiliation for the twelfth author is incorrect. Erica L. T. van den Akker is not affiliated with #2 and #3 but with #1: Obesity Center CGG, Erasmus MC, University Medical Center Rotterdam, Rotterdam, The Netherlands and #4: Division of Endocrinology, Department of Pediatrics, Erasmus MC-Sophia Children’s Hospital, University Medical Center Rotterdam, Rotterdam, The Netherlands.
