# Supplementary material for: Identifying underlying medical causes of pediatric obesity: Results of a systematic diagnostic approach in a pediatric obesity center
Source: PLoS One. 2020 May 8;15(5):e0232990. doi: 10.1371/journal.pone.0232990 (PMC7209105; doi:10.1371/journal.pone.0232990)
Supplement: S1 Appendix — (DOCX) [file pone.0232990.s001.docx]

**Supplementary appendix to *‘Identifying underlying medical causes of pediatric obesity: results of a systematic diagnostic approach in a pediatric obesity center***

1. **Protocol Obesity Center CGG**
2. **Obesity gene panel sequencing details**
3. **Supplementary table**
   S1 Table. P-value table for differences in clinical features between the genetic obesity disorders group and the lifestyle obesity group
4. **Supplementary appendix references**

**1. Protocol Obesity Center CGG pediatric division**

**Background**
Obesity Center CGG (Dutch: ‘*Centrum Gezond Gewicht’*; English: ‘Centre for Healthy Weight’) is a Dutch multidisciplinary national referral center for diagnostics and personalized treatment for patients with obesity. Since 2015 children and adolescents visiting the outpatient pediatric CGG clinics of the university medical center Erasmus MC-Sophia Children’s Hospital have been included. From 2016 on two collaborating general hospitals (Maasstad Ziekenhuis and Franciscus Gasthuis) have also included patients. In the current study, patients from the general hospital Franciscus Gasthuis were not included in our data analysis, as they did not undergo the complete standardized diagnostic procedure. According to Dutch law, written informed consent was obtained from parents and children >12 years; for children below age 12 years oral assent was obtained. This also included separate consent forms for genetic testing.

**Overview of the pathway of the pediatric division of obesity center CGG**

1. Review of historical/referral data
2. Intake by pediatric endocrinologist
3. Anthropometric measurements and vital signs
4. Questionnaires
5. Physiotherapist consultation (only for patients at the academic center Erasmus MC-Sophia Children’s Hospital)
6. Nutritional assessment (only for patients at the academic center Erasmus MC-Sophia Children’s Hospital)
7. Biochemical and hormonal evaluation
8. Genetic testing
9. Development and implementation of the care plan
10. Evaluation of the care plan (follow-up after 1 year)
11. **Review of historical/referral data**

Based on information provided in the referral letter, the patient is referred to the outpatient clinic of the academic center Erasmus MC-Sophia Children’s Hospital (referral indications: suspicion of an underlying cause of obesity including genetic causes of obesity, complex medical history and obesity) or general hospitals Maasstad Ziekenhuis/Franciscus Gasthuis (referral indications: diagnostic evaluation of possible underlying causes as well as comorbidities of obesity, personalized therapeutic advice for non-genetic or non-cerebral causes of obesity, or participation in a combined lifestyle program).^1^ When a patient referred to a general hospital required specific academic expertise, the protocol is completed at the academic center.

1. **Intake by pediatric endocrinologist**

All patients are seen by a pediatric endocrinologist or a pediatrician supervised by a pediatric endocrinologist. Extensive phenotyping is performed to identify underlying endocrine, genetic, cerebral, and medication-induced main causes of obesity. A complete medical history is taken according to the Dutch pediatric guideline for evaluation of children and adolescents with obesity, which includes evaluation of neonatal feeding behavior, current and past weight-inducing medication use, motor and intellectual development, dysmorphic features or congenital anomalies.^2^ This intake visit is not only focused on possible underlying causes of obesity, but also evaluates general health and well-being, lifestyle factors influencing obesity, possible comorbidities, psychosocial circumstances, and other potential barriers for successful treatment.

1. **Anthropometric measurements and vital signs**

Physical examination is performed according to the Dutch guidelines on pediatric obesity.^2^ A wall-mounted stadiometer is used to measure height in 0·1 cm increments. When a child is under the age of two years, recumbent length is measured using an infantometer. Sitting height is the vertical distance between the sitting surface and the top of the head. It is measured in 0·1 cm increments, using the wall-mounted stadiometer and the sitting surface. Weight is measured using a calibrated scale while the children are lightly clothed and standing without shoes. Body mass index (BMI) is calculated as weight/height in meters squared (kg/m^2^). Parental height and weight are also measured when parents are present during the visit at the outpatient clinic; if not present, estimated height and weight of the parents are recorded. Waist circumference in centimeters (0·1 cm increments) is measured between the superior anterior iliac crest and below the lowest rib after normal expiration, with patients standing and unclothed. Occiptofrontal circumference (head circumference; HC) is measured where the largest measurement can be obtained using a flexible tape measure. HC is measured in centimeters (0·1 cm increments). For all measurements, age and sex-specific standard deviation scores (SDS) were calculated using the latest Dutch national growth study as external standard.^3^

Blood pressure is measured on the bared right arm with a digital sphygmomanometer while the patient is seated. Both feet are flat on the floor and the patient is asked not to move or talk during the measurements. Blood pressure is measured twice, the mean is recorded in the patient file. If blood pressure is elevated (>140 mmHg systolic or >90 mmHg diastolic), measurements are repeated twice with short intervals in between. Age, height, and sex-specific standard deviation scores (SDS) are calculated based on the reference values of the American Academy of Pediatrics.^4^ Palpated radial pulse is taken while the patient is seated, registering the number of beats in 30 seconds or digitally assessed by the sphygmomanometer.
All measurements are conducted by outpatient clinic assistants who were specially trained.

1. **Questionnaires**

Patients and/or their parents are asked to fill out the following Dutch questionnaires before or after the visit to the outpatient clinic focusing on physical exercise and fitness, eating behavior, sleep behavior, stress and quality of life:

- Dutch General Obesity Questionnaire^2^
- Dutch Exercise Behavior Questionnaire (in Dutch: ‘*Basis Vragenlijst Bewegen’*, BVB)^5^
- Dutch Eating Behavior Questionnaire (DEBQ)^6^
- Sleep Disturbance Scale for Children (SDSC)^7^
- Perceived Stress Questionnaire (PSQ)^8^
- Pediatric Quality of Life Inventory (PedsQL) 4.0^9^

Data collected through the questionnaires are discussed in the multidisciplinary consultation (see under ‘9. Development and implementation of the care plan’).

1. **Physiotherapist consultation (only for patients at the academic center Erasmus MC-Sophia Children’s Hospital)**

In children and adolescents visiting the outpatient clinic of the academic center Erasmus MC-Sophia Children’s Hospital either the Bruce protocol or the 6-minute walking test (6MWT) is performed under supervision of a pediatric physiotherapist.
The Bruce protocol is a standardized treadmill test with an increasing treadmill speed and incline.^10^ Heart rate and perceived exhaustion are monitored. The test is stopped when the child is exhausted; the maximal endurance time (in minutes, one decimal) serves as criterion of exercise capacity. For children who are not able to perform the Bruce protocol, for example due to intellectual disability, the 6MWT is performed. This test measures how far the patient can walk on a flat track in the exercise room when walking as fast as possible for six minutes. The results of both tests are compared to the norms that have been developed for healthy children.^11-13^ Findings are discussed in the multidisciplinary consultation (see below).

1. **Nutritional assessment (only for patients at the academic center Erasmus MC-Sophia Children’s Hospital)**

The following nutritional assessment is performed for all children and adolescents visiting the outpatient clinic of the academic center Erasmus MC-Sophia Children’s Hospital under supervision of a pediatric dietitian.

- Dietetics: patients or their parents are asked to complete a food diary, recording all foods and drinks consumed over 2 consecutive days. An estimation of the total daily calorie intake is made, as well as an assessment of eating patterns, portion sizes, dietary behavior, and micronutrient intake.
- Resting energy expenditure is measured by indirect calorimetry (Quark RMR, COSMED).
- Body composition (fat mass and fat-free mass) is measured by air displacement plethysmography (BOD POD, COSMED) and/or dual energy x-ray absorptiometry (DEXA).

Findings are discussed in the multidisciplinary consultation (see below).

1. **Biochemical and hormonal evaluation**

Peripheral blood for biochemical and hormonal evaluation is obtained following overnight fasting. Next, a standard oral glucose tolerance test (OGTT) of 1·75 g of glucose per kg body weight (maximum 75 g glucose in 200 ml water) is performed between 8am and 10am. Plasma glucose and insulin are measured at t=0 and at t=2 hours; insulin at t=2 hours is only measured for patients at the academic hospital. The homeostatic model assessment of insulin resistance (HOMA-IR) value is calculated, using a cut-off for insulin resistance of >3·16.^14^ Additionally, at t=0 hemoglobin A1c (HbA1c), total cholesterol, low density lipoprotein (LDL) cholesterol, high density lipoprotein (HDL) cholesterol, triglycerides, alanine transaminase (ALAT), aspartate transaminase (ASAT), Gamma-Glutamyl Transferase (GGT), thyroid hormones (FT4, TSH), cortisol, leptin, insulin-like growth factor 1 (IGF-1), testosterone, anti-Müllerian Hormone (AMH), sex hormone-binding globulin (SHBG), androstenedione, dehydroepiandrosterone sulfate (DHEAS) and 25-hydroxyvitamin D are measured according to local lab standards. All blood analyses are performed at the local medical laboratories of participating hospitals, all of which are ISO 15189 accredited.

1. **Genetic testing**

The following genetic tests are included in the extensive diagnostic workup:

- Next-generation sequencing analysis of obesity associated gene panel
- SNP-microarray analysis

On clinical suspicion, specific additional diagnostic tests (e.g., Prader-Willi syndrome diagnostics, maternal uniparental disomy (UPD) 14 test, trio whole exome sequencing) are performed.
Further details on the genetic tests can be found in the supplemental paragraph 2 ‘Obesity gene panel sequencing details’.

1. **Development and implementation of the care plan**

At the academic center Erasmus MC-Sophia Children’s Hospital, all relevant findings of the diagnostic workup are discussed in a multidisciplinary consultation featuring a pediatric dietitian, a pediatric physiotherapist, pedagogue and pediatric endocrinologist. In this multidisciplinary meeting, the patient-tailored care plan is developed. The care plan includes dietary and physical activity advice, medical treatment (e.g. regarding comorbidities) or referral to combined lifestyle intervention, parent support center, psychologist or psychiatrist. Subsequently, patients are invited to the outpatient clinic to discuss the findings and the care plan. Afterwards, the care plan is communicated to the patient’s referrer, who is responsible for implementing the tailored treatment advices locally.

1. **Evaluation of the care plan (follow-up after 1 year)**

The follow-up visit takes place after at least 1 year and includes evaluation of the patient-tailored care plan during the past year, followed by the same questionnaires, anthropometric measurements, and biochemical and hormonal evaluations (excl. OGTT) as during the intake visit. The results of genetic testing are discussed at the follow-up visit, or earlier when a relevant genetic alteration is found that requires counseling by a clinical geneticist.

**2. Obesity gene panel sequencing details**

Obesity gene panel testing is offered to all children who are included in this study. Because of logistic reasons, there were three different tests available in The Netherlands in the time span of this study. The details of the three obesity gene panels are listed below. The identified variants were compared with in-house and public databases, including [www.mc4r.org.uk](http://www.mc4r.org.uk), to exclude common neutral variants. All variants were analyzed using mutation interpretation software to investigate their (possible) clinical relevance . Variants were classified according to the guideline of The American College of Medical Genetics and Genomics (ACMG).^15^ If possible, a variant of uncertain significance (VUS) or an unknown copy number variation (CNV) was further investigated by family segregation analysis to clarify the pathogenicity. GRCh37/hg19 was used as reference genome.

Obesity Gene panel UMC Utrecht (Department of Genetics, UMC Utrecht, The Netherlands, ISO15189 accredited). December 2014 – November 2016

| **Gene** | **OMIM-entry** | **Inheritance** | **Name of associated syndrome or further details about the disease association** |
| --- | --- | --- | --- |
| *ALMS1* | 606844 | Autosomal recessive | Alstrom syndrome |
| *ARL6* | 608845 | Autosomal recessive | Bardet-Biedl syndrome |
| *BBS1,*  *BBS2,*  *BBS4,*  *BBS5,*  *BBS7,*  *BBS9,*  *BBS10,*  *BBS12* | 209901  606151  600374  603650  607590  607968  610148  610683 | Autosomal recessive | Bardet-Biedl syndrome |
| *BDNF* | 113505 | Autosomal dominant | Obesity associated gene |
| *CCDC28B* | 610162 | Autosomal recessive | Bardet-Biedl syndrome |
| *CEP290* | 610142 | Autosomal recessive | Bardet-Biedl syndrome, Joubert syndrome, Meckel syndrome |
| *CRHR2* | 602034 | - | Corticotropin-releasing hormone receptor |
| *FLOT1* | 606998 |  | Link to cholesterol uptake |
| *G6PC* | 613742 | Autosomal recessive | Glycogen storage disease 1a, von Gierke disease |
| *GNAS* | 139320 | Autosomal dominant | Albright hereditary osteodystrophy |
| *IRS1* | 147545 | Autosomal dominant | Comorbidity gene: insulin receptor |
| *IRS2* | 600797 | Autosomal dominant | Comorbidity gene: insulin receptor |
| *IRS4* | 300904 |  | Comorbidity gene: insulin receptor |
| *KIDINS220* | 615759 | Autosomal dominant | SINO syndrome (spastic paraplegia, intellectual disability, nystagmus, obesity) |
| *LEP* | 164160 | Autosomal recessive | Leptin deficiency |
| *LEPR* | 601007 | Severe: autosomal recessive | Leptin receptor deficiency |
| *LZTFL1* | 606568 | Autosomal recessive | Bardet-Biedl syndrome, Joubert syndrome, Meckel syndrome |
| *MAGEL2* | 605283 | Autosomal dominant | Schaaf-Yang syndrome |
| *MC3R* | 155540 | Autosomal dominant | Obesity associated gene |
| *MC4R* | 155541 | Severe: autosomal recessive  Moderate: autosomal dominant | Melanocortin 4 receptor deficiency |
| *MCHR1* | 601751 |  | Obesity associated gene |
| *MKKS* | 604896 | Autosomal recessive | Bardet-Biedl syndrome, McKusick-Kaufman syndrome |
| *MKRN3* | 603856 | Autosomal dominant | Precocious puberty, Prader-Willi region |
| *MKS1* | 609883 | Autosomal recessive | Bardet-Biedl syndrome, Joubert syndrome, Meckel syndrome |
| *MRAP2* | 615410 | Autosomal dominant | Obesity associated gene |
| *NDN* | 602117 | Isolated cases | Prader-Willi region |
| *NTRK2* | 600456 | Autosomal dominant | Obesity associated gene |
| *PAX6* | 607108 | Autosomal dominant | Aniridia and obesity |
| *PCK1* | 614168 | Autosomal recessive | Phosphoenolpyruvate carboxykinase deficiency, cytosolic |
| *PCSK1* | 162150 | Severe: autosomal recessive  Moderate: autosomal dominant | Obesity with impaired prohormone processing |
| *PHF6* | 300414 | X-linked recessive | Borjeson-Forssman-Lehmann syndrome |
| *POMC* | 176830 | Severe: autosomal recessive  Moderate: autosomal dominant | Obesity, adrenal insufficiency, and red hair due to POMC deficiency |
| *PRKAR1A* | 188830 | Autosomal dominant | Acrodysostosis 1, with or without hormone resistance  Carney complex, type 1  Myxoma, intracardiac  Pigmented nodular adrenocortical disease |
| *PTEN* | 601728 | Autosomal dominant | PTEN hamartoma tumor syndrome |
| *SIM1* | 603128 | Autosomal dominant | Obesity associated gene |
| *SNRPD2* | 601061 | - | Obesity pathway gene |
| *SNRPN* | 182279 | Autosomal dominant | Prader-Willi region |
| *SPG11* | 610844 | Autosomal recessive | Spastic paraplegia 11 |
| *TBX3* | 601621 | Autosomal dominant | Ulnar-mammary syndrome |
| *THRB* | 190160 | Autosomal dominant | Comorbidity gene: thyroid hormone receptor |
| *TMEM67* | 609884 | Autosomal recessive | COACH syndrome, Joubert syndrome Meckel syndrome, Nephronophtisis, modifier of Bardet Biedl syndrome |
| *TRIM32* | 602290 | Autosomal recessive | Bardet Biedl syndrome, Muscular dystrophy, limb girdle, autosomal recessive |
| *TTC8* | 608132 | Autosomal recessive | Bardet Biedl syndrome |
| *TUB* | 601197 | Autosomal recessive | Retinal dystrophy and obesity |
| *WDPCP* | 613580 | Autosomal recessive | Bardet Biedl syndrome |

Next Generation Sequencing (NGS) was performed on a SOLiD 5500XL system (Life Technologies). Horizontal coverage of >99% was achieved. Because of low coverage in a part of the *POMC* gene, additional Sanger sequencing was performed for this gene to achieve >99% horizontal coverage. Further details are provided in Kleinendorst et al., 2018.^16^

Obesity Gene Panel VUmc (Department of Genetics, Amsterdam UMC, location VUmc, The Netherlands, ISO15189 accredited). November 2016 – March 2018
Exome sequencing test with a custom filter. Whole-exome capture was performed using SeqCap EZ MedExome (Roche NimbleGen). Sequencing was done on a HiSeq 2500 or Hiseq 4000 sequencer (Illumina) (paired-end 125 bp and 150 bp reads respectively).The analysis was restricted to variants in a predetermined virtual panel of 52 genes associated with obesity and comorbidities. These were the same 52 genes as in the Utrecht obesity gene panel. If the coverage of the *MC4R* gene was less than 30X, additional Sanger sequencing was performed.

Obesity Gene Panel AMC (Department of Genetics, Amsterdam UMC, location AMC, The Netherlands, ISO15189 accredited). March 2018 - present (inclusion for this study: August 2018)
Gene list: *ALMS1, BDNF, CPE, GNAS, LEP, LEPR, MAGEL2, MC3R, MC4R, NPY4R, PCSK1, PHF6, POMC, SH2B1, SIM1, and VPS13B.*

Targeted enrichment was performed with custom in solution captures (SeqCap EZ Choice, Nimblegen). Sequencing was done on a MiSeq sequencer (Illumina) (paired-end 150 bp reads). All genes had a coverage of >30X. The analysis included CNV detection based on the NGS data. Sequences on chromosome 16p11.2 were included on the capture to allow for detection of a 16p11.2 deletion.

**3. Supplementary table**S1 Table. P-value table for differences in clinical features between the genetic obesity disorders group and the patients without a singular underlying medical diagnosis

|  |  | **Genetic obesity disorders without ID**  **n=19** | **Genetic obesity disorders with ID**  **n=18** | **Total genetic obesity disorders group**  **n=37** | **Total no definite singular underlying medical diagnosis group**  **n=228** | **P-value genetic without ID vs no definite singular underlying medical diagnosis** | **P-value genetic with ID vs no definite singular underlying medical diagnosis** | **P-value total genetic vs no definite singular underlying medical diagnosis** |
| --- | --- | --- | --- | --- | --- | --- | --- | --- |
| **Age at initial visit** | Median (IQR)  [max] | 10.0 (2.9-14.6) [17.7] | 11.2 (7.1-14.7) [16.3] | 10.0 (6.0-14.6) [17.7] | 10.7 (7.7-13.6) [18.0] | P=0.32 (3) | P=0.81 (3) | P=0.36 (3) |
| **Female** | n (%) | 14/19 (74%) | 12/18 (67%) | 26/37 (70%) | 129/228 (57%) | P=0.15 (1) | P=0.41 (1) | P=0.12 (1) |
| **Early-onset <5 years** | n (%) | 18/19 (95%) | 12/18 (67%) | 30/37 (81%) | 146/228 (64%) | **P=0.006 (1)** | P=0.82 (1) | **P=0.04 (1)** |
| **Hyperphagia** | n (%) | 15/19 (79%) | 9/18 (50%) | 24/37 (65%) | 84/228 (37%) | **P<0.001 (1)** | P=0.27 (1) | **P=0.001 (1)** |
| **Height SDS** | Mean (SD) [max] | +1.1 (1.4) [+4.2] | -0.4 (1.3) [+1.5] | +0.3 (1.5) [+4.2] | +0.6 (1.3) [+3.7] | P=0.19 (4) | **P=0.004 (4)** | P=0.36 (3) |
| **Weight SDS** | Mean (SD) [max] | +4.6 (1.5) [+7.0] | +2.3 (1.5) [+5.2] | +3.5 (1.9) [+7.0] | +3.8 (1.1) [+7.1] | **P=0.04 (4)** | **P<0.001 (4)** | P=0.29 (3) |
| **BMI SDS** | Median (IQR)  [max] | +4.2 (+3.5 - +4.7)  [+8.9] | +3.1 (+2.4 - +3.5)  [+5.5] | +3.5 (+2.8 - +4.4)  [+8.9] | +3.8 (+3.3 - +4.3) [+6.6] | P=0.09 (4) | **P<0.001 (4)** | P=0.52 (3) |
| **Head circumference SDS** | Mean (SD) [max] | +2.0 (1.2) [+3.9] | +0.9 (1.5) [+3.8] | +1.4 (1.5) [+3.9] | +1.4 (1.1) [+4.9] | P=0.09 (4) | P=0.20 (4) | P=1.00 (3) |
| **History of neonatal feeding problems** | n (%) | 0/19 | 5/18 (28%) | 5/37 (14%) | 11/228 (5) | P=1.00 (2) | **P=0.003 (3)** | P=0.06 (2) |
| **ID** | n (%) | 0/19 | 12/18 (67%) | 12/37 (32%) | 48/228 (21%) | **P=0.03 (2)** | **P<0.001 (2)** | P=0.13 (1) |
| **Autism** | n (%) | 1/19 (5%) | 2/18 (11%) | 3/37 (8%) | 32/228 (14%) | P=0.48 (2) | P=1.00 (2) | P=0.44 (2) |
| **Parents with obesity** | n (%) | 10/19 (53%)  of which 1 both | 9/18 (50%) | 19/37 (51%) of which 1 both | 161/228 (70%) of which 66 both | P=0.10 (1) | P=0.07 (1) | **P=0.02 (1)** |
| **Parents with history of bariatric surgery** | n (%) | 1/19 (5%)  1 M | 1/18 (6%)  1 M | 2/37 (5%) | 30/228 (13%) of which 3 both | P=0.48 (2) | P=0.71 (2) | P=0.28 (2) |
| **Consanguinity** | n (%) | 2/19 (11%) | 0/18 | 2/37 (5%) | 20/228 (9%) | P=0.68 (2) | P=0.38 (2) | P=0.75 (2) |
| **Psychosocial problems** | n (%) | 3/19 (16%) | 4/18 (22%) | 7/37 (19%) | 115/228 (50%) | **P=0.004 (1)** | **P=0.02 (1)** | **P=0.001 (1)** |
| **Current/past use of weight-inducing medication** | n (%) | 5/19 (26%) | 2/18 (11%) | 7/37 (19%) | 59/228 (26%) | P=1.00 (2) | P=0.26 (2) | P=0.36 (1) |
| **Evidently dysmorphic appearance and/or congenital anomaly** | n (%) | 1/19 (5%) | 12/18 (67%) | 13/37 (35%) | 32/228 (11%) | P=0.48 (2) | **P<0.001 (2)** | **P=0.002 (1)** |
| **Lifestyle factors as most important contributor to obesity** | n (%) | 1/19 (5%) | 0/18 | 1/37 (3%) | 72/228 (32%) | **P=0.02 (1)** | **P=0.005 (1)** | **P<0.001 (1)** |
| **Socio-economic status z-score** | Median (IQR) [min] | 0.0 (-1.0 - +0.5)  [-2.6] | -0.3 (-1.2 - +0.3)  [-1.8] | 0.0 (-1.0 - +0.4)  [-2.6] | -0.1 (-1.4 - +0.5) [-4.8] | P=0.76 (4) | P=0.95 (4) | P=0.59 (3) |
| **Short stature** | n (%) | 0/19 | 4/18 (22%) | 4/37 (11%) | 7/228 (3%) | P=1.00 (2) | **P=0.005 (2)** | P=0.052 (2) |
| **Tall stature** | n (%) | 6/19 (32%) | 1/18 (6%) | 7/37 (19%) | 53/228 (22%) | P=0.41 (2) | P=0.13 (2) | P=0.56 (1) |
| ID, intellectual disability; IQR, interquartile range; max, maximum; SDS, standard deviation score; M, mother. (1) Chi squared test; (2) Fisher’s exact test; (3) Independent sample t-test (if necessary, after log transformation). (4) Mann-Whitney U test. Cells in bold indicate a statistically significant difference between the mentioned groups. | | | | | | | | |

**4. Supplementary appendix references**

1. de Niet J, Timman R, Jongejan M, Passchier J, van den Akker E. Predictors of participant dropout at various stages of a pediatric lifestyle program. *Pediatrics* 2011; **127**(1): e164-70.

2. Van den Akker ELT, Vreugdenhil A, Hustinx SR, Verkaaik M, Houdijk ECAM, Van Mil E. Obesity in children and adolescents: guideline for pediatricians (Dutch*: "Obesitas bij kinderen en adolescenten: Leidraad voor kinderartsen*") 01-08-2018. https://www.nvk.nl/Kwaliteit/Richtlijnen-overzicht/Details/articleType/ArticleView/articleId/2066/Obesitas-leidraad-voor-kinderartsen-2018 (accessed 12-06-2018).

3. Schonbeck Y, Talma H, van Dommelen P, et al. Increase in prevalence of overweight in Dutch children and adolescents: a comparison of nationwide growth studies in 1980, 1997 and 2009. *PLoS One* 2011; **6**(11): e27608.

4. Flynn JT, Kaelber DC, Baker-Smith CM, et al. Clinical Practice Guideline for Screening and Management of High Blood Pressure in Children and Adolescents. *Pediatrics* 2017; **140**(3).

5. Van Wieringen JCM. *Standpunt Beweegstimulering door de jeugdgezondheidszorg* [English: Exercise stimulation by the pediatric public health service]. Rijksinstituut voor Volksgezondheid en Milieu (RIVM) [National Institute for Public Health and the Environment]. Bilthoven, The Netherlands: Dutch Ministry of Health, Welfare and Sport; 2009. p. 45-53. Available from <https://www.rivm.nl/bibliotheek/rapporten/295002001.pdf>. Accessed 2019-01-15.

6. Van Strien T, Rookus MA, Bergers GP, Frijters JE, Defares PB. Life events, emotional eating and change in body mass index. *Int J Obes* 1986; **10**(1): 29-35.

7. Bruni O, Ottaviano S, Guidetti V, et al. The Sleep Disturbance Scale for Children (SDSC). Construction and validation of an instrument to evaluate sleep disturbances in childhood and adolescence. *J Sleep Res* 1996; **5**(4): 251-61.

8. Levenstein S, Prantera C, Varvo V, et al. Development of the Perceived Stress Questionnaire: a new tool for psychosomatic research. *J Psychosom Res* 1993; **37**(1): 19-32.

9. Varni JW, Seid M, Kurtin PS. PedsQL 4.0: reliability and validity of the Pediatric Quality of Life Inventory version 4.0 generic core scales in healthy and patient populations. *Med Care* 2001; **39**(8): 800-12.

10. Bruce RA, Blackmon JR, Jones JW, Strait G. Exercising Testing in Adult Normal Subjects and Cardiac Patients. *Pediatrics* 1963; **32**: SUPPL 742-56.

11. Geiger R, Strasak A, Treml B, et al. Six-minute walk test in children and adolescents. *J Pediatr* 2007; **150**(4): 395-9, 9 e1-2.

12. van der Cammen-van Zijp MH, Ijsselstijn H, Takken T, et al. Exercise testing of pre-school children using the Bruce treadmill protocol: new reference values. *Eur J Appl Physiol* 2010; **108**(2): 393-9.

13. van der Cammen-van Zijp MH, van den Berg-Emons RJ, Willemsen SP, Stam HJ, Tibboel D, H IJ. Exercise capacity in Dutch children: new reference values for the Bruce treadmill protocol. *Scand J Med Sci Sports* 2010; **20**(1): e130-6.

14. Keskin M, Kurtoglu S, Kendirci M, Atabek ME, Yazici C. Homeostasis model assessment is more reliable than the fasting glucose/insulin ratio and quantitative insulin sensitivity check index for assessing insulin resistance among obese children and adolescents. *Pediatrics* 2005; **115**(4): e500-3.

15. Richards S, Aziz N, Bale S, et al. Standards and guidelines for the interpretation of sequence variants: a joint consensus recommendation of the American College of Medical Genetics and Genomics and the Association for Molecular Pathology. *Genet Med* 2015; **17**(5): 405-24.

16. Kleinendorst L, Massink MPG, Cooiman MI, et al. Genetic obesity: next-generation sequencing results of 1230 patients with obesity. *J Med Genet* 2018; **55**(9): 578-86.
